# Stellate ganglion block with procaine in breast cancer survivors with hot flashes and sleep disturbances undergoing Endocrine Therapy

**DOI:** 10.1097/MD.0000000000036848

**Published:** 2024-01-12

**Authors:** Sven Soecknick

**Affiliations:** a Neural Therapy and Acupuncture, Luebeck, Germany.

**Keywords:** breast cancer, hot flashes, neural therapy, procaine, Stellate ganglion Block

## Abstract

Breast cancer survivors under endocrine therapy (ET) suffer from side effects such as hot flashes and sleep disturbance accompanied by poor quality of life. Many quit ET early and reduce their survival rate. Guidelines recommend gabapentin next to yoga or acupuncture. The role of side effects related to compliance with ET over years require new and effective therapies. Stellate ganglion block (SGB) has shown evidence of safety and efficacy and was found to be more effective than pregabalin without side effects. However, practical guidelines for the long-term use of SGB are still missing. We primarily used procaine instead of bupivacaine presuming effectiveness paired with lower toxic risks. Twenty-nine breast cancer survivors with severe hot flashes and sleep disturbance under ET received SGB with Procaine. Diaries recorded hot flashes and sleep quality scores up to week 24. All patients took part and none refused SGB. Each Patient received one SGB every 4 weeks without any side effects observed. Weekly scores were reduced from baseline by −33.6% (*P* < .01) (hot flashes) and −22.3% (*P* < .01) (sleep disturbances) after 4, and by −58.8% (*P* < .01) (hot flashes) and −50.8% (*P* < .01) (sleep disturbances) after twenty-for weeks. A wavelike reduction indicated a limited effect of a single SGB during continuous ET. We showed, that procaine in SGB is as effective as bupivacaine with lower risks and costs. High significant reductions in hot flashes and sleep disturbances after 1 and 6 months were found. We conclude that breast cancer survivors need individual treatment with SGB due to her personal impact. Hence, SGB should find its way to guidelines and daily routines as a valuable method for treating side effects in breast cancer survivors undergoing ET.

## 1. Introduction

Breast cancer is the most common type of carcinoma among women. As 75% of breast cancer survivors are hormone-responsive, adjuvant (ET) remains the standard treatment following surgery, adjuvant chemotherapy, and radiation for these patients. Current guidelines suggest, a 5 to 10 years ET with Tamoxifen or Aromatase Inhibitors at the highest level (++).^[1]^

Although Tamoxifen is well known for its efficacy and toxicity^[2]^ and has been used for decades, its side effects often limit its benefits. The most common side effects attributed to ET are hot flashes (64%), vaginal dryness (35%), and sleep disturbances (36%).^[3,4]^ Aromatase inhibitors (AI) also cause hot flashes and sleep disturbances at similar rates following their main adverse effects, such as osteoporosis, cardiovascular risks, and musculoskeletal symptoms,^[4]^ although different trials have shown significantly lower rates.^[5]^ Younger online requests^[6]^ showed higher side effects if patients were asked directly.

In summary, ET can lower the risk of breast cancer recurrence significantly.

However, despite the proven effects of ET, the risk of recurrence is the highest during the first 3 years after the initial diagnosis and treatment. 36% of breast cancer survivors quit ET early (between 25 months and 4.1 years) because of the side effects of medications.^[7]^ Partridge et al^[8]^ reported an overall decrease of 50% in the 4th year of therapy. Therefore, underestimation of residual side effects by physicians from recent chemotherapy or radiation therapy when starting ET seems to play an important role. Regular follow-ups, in which patients can freely express their perceptions, beliefs and suffering, seem to significantly improve adherence to ET.^[9]^

Otherwise, sufficient simultaneous treatment against the side effects of ET is needed. It is as important as ET itself, because it represents the greatest reason for the discontinuation of ET.^[10,11]^

Regarding hot flushes and sleep disturbances, current guidelines recommend venlafaxine, selective serotonine reuptake inhibitors (SSRI), or gabapentin for treatment of hot flushes and sleep disturbances.^[1]^ However, these drugs often produce significant additional side effects. Therefore, patients tend to refuse these possibilities. Moderate results were obtained for acupuncture Stellate ganglion Block^[12]^ and yoga.^[13]^

Owing to the special role of side effects related to compliance with ET for 5-10 years more, other effective therapies are required. With increasing understanding of the pathophysiology, hot flashes, sleep disturbances, and vasomotor symptoms appear to occur in response to rapidly diminishing estrogen production.^[14–16]^ Lipov et al not only presented a neurophysiological theory for hot flashes.^[17]^ They postulated a deactivated norepinephrine pathway in the brain caused by reduced concentrations of nerve growth factor due to tamoxifen. They also showed, that Stellate ganglion Block (SGB) can be a sufficient therapy to relieve hot flashes by interrupting the sympathetic nervous system.^[18–20]^ Haest et al^[21]^ also considered the reason for hot flashes in thermoregulatory dysfunction within the hypothalamus triggered by estrogen withdrawal. They appointed SGB as an effective treatment for breast cancer survivors with vasomotor symptoms and/or sleep disturbances. Othman et al^[22]^ showed that SGB has superior efficacy in the management of hot flashes in breast cancer survivors compared with pregabalin.

SGB, used for decades as a safe and effective method for different indications, has proven to be a safer and more effective treatment for hot flashes and sleep disturbance in breast cancer survivors than current pharmacological alternatives.^[18,19,21,23]^ However, large prospective randomized controlled trials for SGB in hot flashes and sleep disturbances are still lacking.^[24]^

In previous studies on SGB in breast cancer survivors known by the author, all patients underwent SGB with bupivacaine under real-time fluoroscopic imaging using contrast dye. The most common reason for this safety procedure might be founded in the highest toxicity of bupivacaine comparing LA not only intravasal, but also in increased periinjectional resorption, especially cardiac arrhythmia can occur also in the absence of central nervous system (CNS) side effects.

Owing to the varying intensities of blockade by different local anesthetics (LA) in nerve tissues, high-frequency nociceptive afferences (“firing nerves”) are much easier to block with lower doses of LA than low-frequency motor neurons or tactile afferences.^[25]^

Based on this awareness, we suppose that not the duration of LA, needed in surgical situations, does the positive effect, but interrupting the negative sympathetic neuronal feedback loops.

The aim of our clinical study was to identify a practical, long-lasting and usable treatment of side effects such as hot flashes and sleep disturbances in breast cancer survivors undergoing ET using SGB. In our opinion, to achieve this aim, an easy but safe way of executing SGB, a short-acting local anesthetic with comparable efficacy to bupivacaine and a simple method of documentation of daily side effects are needed to obtain an acceptable cost-effectiveness ratio as well as an accepted procedure by patients.

## 2. Patients and methods

### 2.1. Study design and setting

This study was a single-center, uncontrolled trial of female breast cancer survivors as a treatment for hot flashes and sleep dysfunction with SGB. Patients were referred by their gynecologists or oncologists. All suffered from severe life-limiting hot flashes and sleep disturbances, and were enrolled at the physician’s office of the author (Luebeck, Germany) between April 2014 and September 2017. The participation was elective. Written informed consent, even for the study design and SGB treatment was obtained from all patients. Patients with changes in ET less than 8 weeks ago, Karnofski´s index under 80, severe cardiac disorders, severe acute infection, use of anticoagulants (except low-dose aspirin), acute therapy with SSRI or gabapentin and phobia against syringes were excluded from the study.

### 2.2. Participants

29 breast cancer survivors were included in the study. All the patients underwent surgery and additional chemotherapy and/or radiation therapy. All the patients underwent ET with either Tamoxifen or AI because of their hormone receptor-positive status. None of the patients had contraindications.

**Figure 1.**
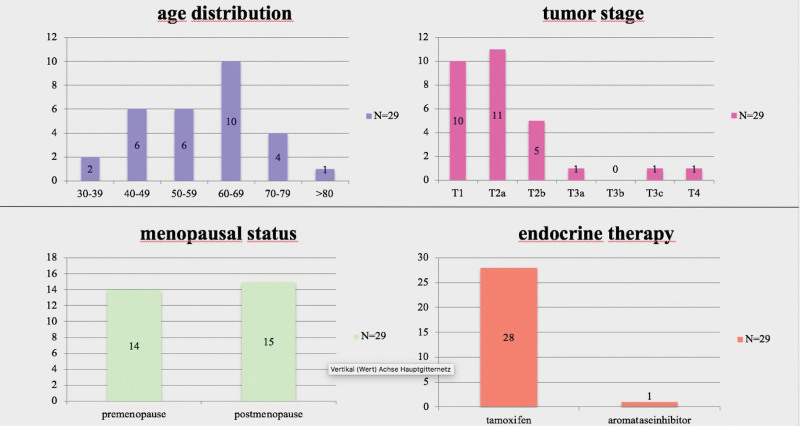
Patients spreading in age, tumor stage, menopausal status, endocrine therapy.

The age distribution, tumor stage, menopausal status, and type of ET are shown in Figure 1.

### 2.3. Measurements

Self-completed daily diaries were used to collect data, as validated by Sloan et al^[26]^ and has been used in other hot-flash studies, such as that by Haest et al^[21]^ Therefore, a weekly hot flash score was calculated (frequency multiplied by average severity (0 = none, 1 = mild, 2 = moderate, 3 = severe) multiplied by 7 days) to reduce daily fluctuations in sweating due to short-term influences other than ET.

To measure sleep quality according to the Pittsburgh Sleep Quality Index (PSQI), patients had to estimate themselves on a scale from 0 to 3 (0 = no sleep disturbance (SD), 1 = low SD, 2 = moderate SD, 3 = severe SD) in the morning. The sleep quality index was calculated on a weekly basis.

### 2.4. Materials

Every SGB injection was performed with procaine 1% 5 mL.

Due to the aim of the trial, if other LA show similar effects on hot flashes and sleep quality like bupivacaine, we used procaine 1%. Procaine is a well-known short-term LA with a duration of up to 30 minutes. Its active metabolites (para-amino-benzoe-acid-PABA and diethylaminoethanol DAEA) produce vasodilatory, anti-inflammatory and membrane-stabilizing effects. As used in neural therapy (NT and pain therapy since decades, the real purpose of short-term LA like Procaine is not to achieve local anesthesia^[17,27]^ but to achieve reorganization of the nervous system, tissue perfusion, anti-inflammation and disruption of negative feedback actions (vicious circle).^[28]^

Relative fat solubility of procaine is, in comparison to bupivacaine, a thousand-fold lower (0.02 vs 141)^[25]^ causing lower cardiac risk in case of malinjection. The frequently mentioned high allergy potential in the past is not comprehensible from a new perspective.^[25]^

### 2.5. Stellate ganglion block (SGB)

Before each injection, the physician (author) checked whether none of the exclusion criteria had been met. The physician is well-educated and experienced in treating LA while practicing over 10 years of neural therapy for the treatment of pain and other diseases. The author owns the education certificate of the international society of Huneke’s Neuraltherapy since 2008.

For SGB, the technique described by Leriche (1985) and Fontaine and Dosch (1986), modified by Fischer,^[29]^ was used as described in detail in 2016.^[27]^ In Comparison with the techniques mostly used by anesthesiologists, lateral access to the Chassaignac´s tubercle is preferred. Therefore, the neurovascular bundle (common carotid artery, vagus nerve and internal jugular vein) can be shifted medially away from the injection zone. One millimeter below the Chassaignac´s tubercle and after double-negative aspiration for blood and cerebrospinal fluid, 5 mL of procaine 1% was injected slowly. Therefore, the executive physician ensured that none of the injections were deeper than 2 cm. All SGB injections were administered by the author.

The effect of SGB on the sympathetic nervous system was confirmed by the presence of ipsilateral Horner´s syndrome (ptosis, miosis, enophtalmus), blood-shot conjunctiva, and an increase in temperature of the ipsilateral upper extremity. All SGB were performed on the contralateral side of the ill breast (in cases of illness in both breasts, the right side was used). After the injection, oxygen saturation, heart frequency, blood pressure, and patient vigilance were monitored for up to 30 minutes. No side effects were observed for any SGB. No negative hemodynamic effects were observed after any of the SGB injections and no negative hemodynamic effects were observed, as previously proposed.^[27]^

As the positive effect of LA on hot flashes and sleep disturbance has still been shown,^[24,30]^ it does not seem to be suspended after a single SGB but at an early onset,^[21,31]^ we concluded, that under continuous ET, breast cancer survivors suffer from a permanent negative influence of it and therefore need individual continuous treatment with SGB, as Forouzanfar also found longer-lasting effects with repeated SGB.^[32]^

In summary, each patient received SGB every 4 weeks in summary 6 injections in 24 weeks.

### 2.6. Statistical analysis

The analyzed data were collected by daily diaries. The main efficacy measures used in this trial were the mean average of the weekly total hot-flash scores, as explained above and the change in mean average of sleep quality from baseline.

The baseline was determined using a 7-day diary of hot flashes and sleep quality before the first SGB.

Some studies analyzed each level of hot flashes separately and built a total flash score.

Othman et al^[22]^ indicated, that except of mild hot flashes, all others showed very similar movements.

As our aim was to find the most practical way to assess symptoms in a doctor’s practice, we decided to use the total hot flash score to evaluate the progress of hot flashes.

The basis of the analysis for this study was descriptive summary statistics (means, standard deviations, and percentages).

The generalized estimating equation method (GEE) was used, assuming that the conditions for “classical” regression models are not preexisting, and they are to be used in study designs with measurement repetitions within the same patient.^[16,33]^ Wald-Chi2 at the 0.01 level were then used to test the null hypothesis of no treatment. The GENLIN procedure in the statistical program SPSS Ver.24 IBM, Armonk, NY was used for the statistical analysis.

#### 2.6.1. Descriptive statistics total hot flashes.

The longitudinal regression model for hot flashes showed a continuous decrease of 33.6% in the hot flash score after 4 weeks of undergoing the first SGB versus baseline (99% CI). See Table 1.

**Table 1:** Hotflashes over time from week 0 to week 24 (n = 29).

Week	Min	Max	M (mean)	SD	Post hoc-tests	*P* value
					M_week 1_ − M_week 1 + k_	
0	3	256	109.69	66.042	–	–
1	2	289	93.24	70.823	16.45	.024
2	0	251	80.76	65.639	28.93	<.010
3	0	233	65.07	56.270	44.62	<.010
4	2	280	72.86	65.526	36.83	<.010
5	0	257	70.72	65.919	38.97	<.010
6	1	241	62.38	57.721	47.31	<.010
7	1	248	59.86	56.041	49.83	<.010
8	2	231	58.90	54.764	50.79	<.010
9	5	234	61.24	60.292	48.45	<.010
10	1	218	58.90	60.774	50.79	<.010
11	1	299	63.93	68.785	45.76	<.010
12	0	236	63.79	63.001	45.90	<.010
13	0	240	60.55	61.479	49.14	<.010
14	0	266	57.66	64.181	52.03	<.010
15	0	272	50.24	67.941	59.45	<.010
16	0	233	53.10	63.114	56.59	<.010
17	0	216	47.52	50.000	62.17	<.010
18	0	197	53.14	54.173	56.55	<.010
19	0	209	48.90	50.164	60.79	<.010
20	0	209	44.55	52.895	65.14	<.010
21	0	202	48.07	53.901	61.62	<.010
22	0	198	45.10	56.709	64.59	<.010
23	0	233	45.90	59.556	63.79	<.010
24	0	203	45.17	52.088	64.52	<.010

**Figure 2.**
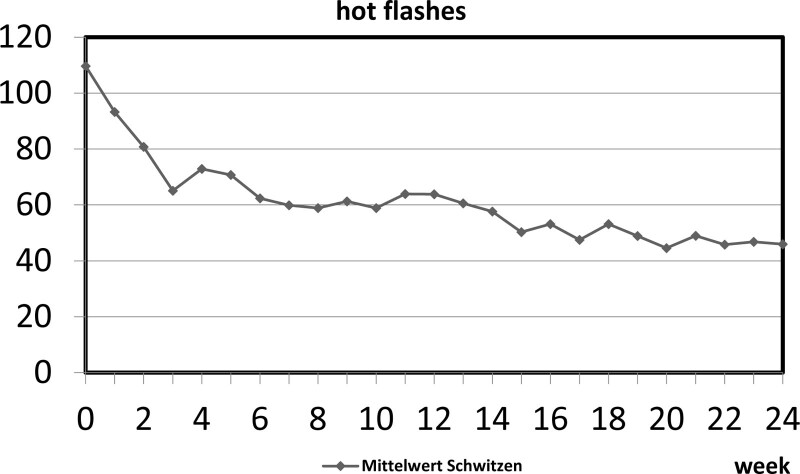
Mean of hot flashes from week 0 to week 24.

Figure 2 shows the progress of the decrease in hot flashes during receiving SGB every 4 weeks down to a reduction of 58.8% (99% CI), having received a total of 6 SGB after 24 weeks.

#### 2.6.2. Descriptive statistics sleep disturbances.

The longitudinal regression model for sleep disturbance showed a continuous reduction of 22.3% after 4 weeks of undergoing the first SGB versus baseline (99% CI). (See Table 2).

**Table 2:** Sleep disturbance over time from week 0 to week 24 (n = 29).

Week	Min	Max	M (mean)	SD	Post hoc-tests	*P* value
					M_week 1_ − w_Wweek1 + k_	
0	4	32	13.66	6.731	–	–
1	0	33	12.59	7.390	1.07	.129
2	0	32	11.52	7.342	2.14	.024
3	0	28	9.76	6.770	3.90	<.010
4	0	31	10.62	7.068	3.03	.002
5	0	31	9.52	6.937	4.14	<.010
6	0	30	9.48	6.854	4.17	<.010
7	0	25	9.10	7.048	4.55	<.010
8	0	26	9.83	6.788	3.83	<.010
9	0	47	9.72	9.877	3.93	.003
10	0	37	9.31	8.177	4.34	<.010
11	0	29	8.17	7.061	5.48	<.010
12	0	32	7.41	8.292	6.24	<.010
13	0	35	7.79	8.113	5.86	<.010
14	0	21	7.59	6.764	6.07	<.010
15	0	21	6.86	5.730	6.79	<.010
16	0	30	7.72	7.657	5.93	<.010
17	0	26	7.14	6.653	6.52	<.010
18	0	25	8.41	7.273	5.24	<.010
19	0	23	7.66	6.119	6.00	<.010
20	0	23	7.03	6.015	6.62	<.010
21	0	26	7.10	6.873	6.55	<.010
22	0	24	6.55	6.711	7.10	<.010
23	0	24	6.52	7.937	7.14	<.010
24	0	23	6.72	6.871	6.93	<.010

**Figure 3.**
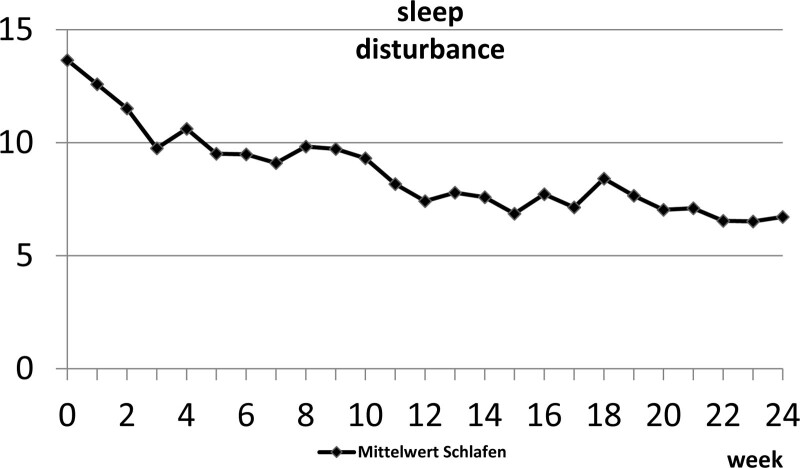
Mean of sleep disturbance from week 0 to week 24.

Figure 3 shows the progress of the decrease in sleep disturbance during SGB every 4 weeks down to a reduction of 50.8% (99% CI), having received a total of 6 SGB sessions after 24 weeks.

## 3. Results

Twenty-nine female breast cancer survivors with sleep disturbances and severe, non-tolerable hot flushes participated in this study. All patients completed the study after 24 weeks, each undergoing a procedure of receiving SGB with 5 mL procaine at the contralateral side of the breast cancer once every 4 weeks. None of the patients reported adverse side effects such as tachycardia, collapse, low oxygen levels or low blood pressure. No additional donation of sedation or local anesthetics other than procaine were required because of the less painful and short-term injection technique used.^[27]^

Figures 2 and 3 show, as other groups report identically, a quite fast decrease in total hot flashes and sleep disturbance, where our data suggest a slower decrease than others.

In all Tables and Figures Baseline is marked as “week 0” indicates the conditions of hot flashes and sleep disturbance before the first therapy.

## 4. Discussion

SGB for the treatment of hot flashes and sleep disturbances in breast cancer survivors has been reported in several studies^[18,21,24]^ and has proven to be better than pharmaceutical therapies^[22]^ without potential systemic side effects, especially with long-term use.

Although official agencies estimate its significance as no longer experimental anymore,^[34]^ SGB has still not reached a level of common use in doctors’ daily practice for the treatment of adverse effects of ET. High technical effort paired with high costs per procedure ranging from US$1.000 up to US$3.000 in the US^[35]^ and missing clinical guidelines for the long-term use of ET^[34]^ seem to be the main obstacles in implementing this therapy.

To the best of our knowledge, this is the first study to use procaine instead of bupivacaine to manage hot flashes and sleep disturbances in breast cancer survivors with SGB. The therapeutic use of procaine in NT has been widespread since its inception in 1925. Although replaced in surgery for a long time by LA with a longer duration, procaine undergoes a kind of renaissance because of its rebutted allergy potential^[25]^ and further information about the mechanism of action in the autonomic nervous system and its additional effects such as anti-inflammatory, membrane stabilization, and boosting of precapillary blood flow.^[28]^

Similar to other studies,^[18,21,22]^ we found SGB to be a safe, effective, and practical method for treating breast cancer survivors who experience hot flashes and sleep disturbances during ET. We presume that the fundamental mechanisms underlying the effects of SGB in treating the side effects of ET as to be evident.

Sloan et al found that 25 patients per trial could provide a reasonable estimation^[26]^; therefore, our trial with 29 patients should have the power to achieve acceptable results. They considered reductions of > 50% to be greater than would be expected with placebo, whereas our data showed a reduction in hot flashes and 58.8% in sleep disturbances of 50.8%.

However, the aim of this study was not only to show whether SGB is better than placebo, what it did, or comparable to oral medication like Othman et al,^[22]^ nor to determine the duration of the effect of a single SGB.^[21]^ Haest et al,^[21]^ who found that a single SGB persists over weeks but increases continuously over 24 weeks, concluded that due to the continuous negative effects of daily oral ET, patients need a kind of intermittent treatment versus those side effects over the period they are treated with ET. This cognition is based on Lipov´s explanation of the pathological mechanisms that produce side effects during ET.^[17]^

Our data showed a wavelike decreasing trend in hot flashes as well as in sleep disturbances.

This indicates a terminated effect on the symptoms of each SGB during the permanent negative effect of ET. Based on this assumption, we presume the need for individual SGB treatments in each patient. This finding bases also on the individual data of each Patient in detail. Although the average of all patients showed significant decreases in hot flashes and sleep disturbances in the short term as well as in the long term, the totality of our patients did not react concordantly. Most patients showed an average approach like Figures 1 and 2 picture it. However, there were also a couple of patients who could not benefit from SGB. Another small group was nearly symptom-free after 3 months and probably did not require more SGB. While pretending an interval of 4 weeks to receive SGB based on the trial protocol, after 6 months, most patients were able to appraise, weather they needed shorter or longer intervals to receive SGB.

As a mean argument against continuous treatment with SGB against hot flashes and sleep disturbances, often high costs of 1000 to 3000 US$ per injection are named.

Anesthesiologists, neural therapists, pain therapists, and others practice SGB in outpatients without fluoroscopy, using anatomical landmarks based on the technique described by Leriche and Dosch and also used by Puente de la Vega Costa.^[27]^ In summary, SGB performed by neural therapists with procaine has been shown to be a practical and safe method, especially because of the much lower cardiotoxicity of procaine in comparison to bupivacaine because of its fat-solubility (procaine 0.02 vs bupivacaine 141) in case of malinjection. From our own calculation, the cost of SGB in a standard outpatient pain clinic may summarize to 100 to 150€ and thereby 10 to 20 times lower than it is estimated by Loprinzi.^[35]^ Under these economic conditions, it is important to recommend SGB to breast cancer survivors under ET who experience hot flashes and sleep disturbances as a valid, safe and effective therapy against side effects to achieve a better quality of life while continuing endocrine therapy as oncologists advise it.

Although 24 weeks seems to be a good observation period, a drawback in determining the individual interval for SGB, a longer period is necessary. Another drawback of our trial is still a minor number of patients. To confirm our findings, a multicenter study with different experienced therapists is required.

## 5. Conclusion

Overall, SGB is a safe, valid and effective method to treat hot flashes and sleep disturbances in breast cancer survivors undergoing ET. It has passed its experimental stadium and shown practical use in the treatment of these side effects. Besides the question of which LA should be used, there is enough data and experience of SGB that oncologists and gynecologists should be informed via national and international guidelines about the possibility of treatment with SGB. Under SGB, breast cancer survivors receive an additional opportunity to treat their side effects and achieve a better quality of life while compliance increases to continue ET, as indicated.

## Acknowledgments

I thank the 29 patients for their participation. I thank MD Jens Kisro, Oncologist, Luebeck, for his oncological advice during this trial. I also thank Mr. Ralf Gruenwald for statistical analysis and assistance. Special thanks to Mrs. Kirsten Meissner, a study nurse, for untiring commitment to collecting patient data.

## Author contributions

**Conceptualization:** Sven Soecknick.

**Project administration:** Sven Soecknick.

**Writing – original draft:** Sven Soecknick.
